# Examining the Effect of ALK and EGFR Mutations on Survival Outcomes in Surgical Lung Brain Metastasis Patients

**DOI:** 10.3390/cancers15194773

**Published:** 2023-09-28

**Authors:** Sneha Sai Mannam, David P. Bray, Chibueze D. Nwagwu, Jim Zhong, Hui-Kuo Shu, Bree Eaton, Lisa Sudmeier, Subir Goyal, Christopher Deibert, Edjah K. Nduom, Jeffrey Olson, Kimberly B. Hoang

**Affiliations:** 1 Department of Neurosurgery, Perelman School of Medicine, University of Pennsylvania, Philadelphia, PA 19104, USA; 2 Department of Neurosurgery, Jefferson University Hospital, Philadelphia, PA 19107, USA; 3 Department of Neurosurgery, Brigham and Women’s Hospital, Boston, MA 02115, USA; 4 Department of Radiation Oncology, Emory University School of Medicine, Atlanta, GA 30322, USAhgshu@emory.edu (H.-K.S.);; 5 Biostatistics Shared Resource, Winship Cancer Institute, Emory University, Atlanta, GA 30322, USA; 6 Department of Neurosurgery, Emory University School of Medicine, Atlanta, GA 30322, USA

**Keywords:** brain metastases, lung cancer, systemic therapies, epidermal growth factor receptor, anaplastic lymphoma kinase, radiosurgery

## Abstract

**Simple Summary:**

In this research study, the authors investigated the impact of specific genetic mutations on the survival of lung cancer patients with brain metastases who underwent surgical resection. These mutations, known as anaplastic lymphoma kinase (ALK)-rearranged and epidermal growth factor receptor (EGFR)-amplified mutations, have shown potential for targeted treatments. The study analyzed data from patients who received surgical treatment at Emory University Hospital between 2012 and 2022. Results showed that the overall survival and progression-free survival rates in this group were better than those seen in earlier studies. The study suggests that as more targeted therapies become available, the survival rates for lung cancer patients with brain metastases may continue to improve. The findings emphasize the importance of individualized treatments based on genetic mutations.

**Abstract:**

In the context of the post-genomic era, where targeted oncological therapies like monoclonal antibodies (mAbs) and tyrosine-kinase inhibitors (TKIs) are gaining prominence, this study investigates whether these therapies can enhance survival for lung carcinoma patients with specific genetic mutations—EGFR-amplified and ALK-rearranged mutations. Prior to this study, no research series had explored how these mutations influence patient survival in cases of surgical lung brain metastases (BMs). Through a multi-site retrospective analysis, the study examined patients who underwent surgical resection for BM arising from primary lung cancer at Emory University Hospital from January 2012 to May 2022. The mutational statuses were determined from brain tissue biopsies, and survival analyses were conducted. Results from 95 patients (average age: 65.8 ± 10.6) showed that while 6.3% had anaplastic lymphoma kinase (ALK)-rearranged mutations and 20.0% had epidermal growth factor receptor (EGFR)-amplified mutations—with 9.5% receiving second-line therapies—these mutations did not significantly correlate with overall survival. Although the sample size of patients receiving targeted therapies was limited, the study highlighted improved overall survival and progression-free survival rates compared to earlier trials, suggesting advancements in systemic lung metastasis treatment. The study suggests that as more targeted therapies emerge, the prospects for increased overall survival and progression-free survival in lung brain metastasis patients will likely improve.

## 1. Introduction

Multimodal treatment of metastatic cancer has changed in the genomic era. Targeted therapies have improved systemic disease control in subsets of metastatic disease [1,2], most notably for breast cancer, lung cancer, and melanoma [3,4,5]. As systemic disease is better controlled and overall survival (OS) of patients with metastatic disease improves, the incidence of brain metastases (BMs) has also risen [5,6,7].

Meanwhile, significant progress has been made in function-sparing microsurgical techniques for the resection of solitary, dominant, and multiple symptomatic BMs [8,9]. Combining microsurgery with radiation therapy in the form of whole brain radiotherapy (WBRT), focused radiation stereotactic radiosurgery (SRS), and fractionated SRS has resulted in local control rates of 80%, 82%, and 84%, respectively [10,11,12,13,14]. New systemic immune-modulating therapies or tyrosine kinase inhibitors (TKIs) have demonstrated clinical evidence of at least partial blood–brain barrier (BBB) penetration and efficacy, unlike prior chemotherapeutic agents [15]. New guidelines from the National Comprehensive Cancer Network (NCCN) now mention the emerging role of systemic therapy in brain disease [16]. Multiple ongoing clinical trials in lung, breast, and melanoma are underway to better elucidate the role of systemic therapy in the multimodal algorithm for brain metastases treatment [17,18,19,20,21,22,23,24,25,26,27,28,29].

The incidence of anaplastic lymphoma kinase (ALK)-rearranged mutations and epidermal growth factor receptor (EGFR)-rearranged mutations in lung cancer patients is around 5% and 15%, respectively. Approximately 20 to 40% of patients with metastatic lung cancer will develop BMs [30,31,32,33]. Among these, 25% of lung cancer patients develop ALK-rearranged mutations [34] and 33% of patients develop activating mutations [35]. Both ALK and EGFR mutations strongly correlate with favorable systemic therapeutic responses to TKIs and increased OS [36,37]. The incidence of BMs in EGFR and ALK patients is also significantly higher than other mutational types [17,38], with approximately 30% of ALK and 40% of EGFR patients having BMs [39,40]. Notably, retrospective, single-institution analysis has suggested that systemic mutational status in patients with melanoma BM correlates to improved local control (LC) and OS in patients undergoing craniotomy for resection [41]. This is significant given that melanoma was one of the first systemic malignancies to demonstrate the cerebral penetrance of systemic therapy and role of molecular drivers (BRAF) in prognostication. The role of mutational status has not been well described in patients with lung BMs.

Therefore, this study investigates the role of mutational status in lung cancer BM patients undergoing surgical resection with respect to LC and OS. With our study, we aim to (1) review the relationship between the extent of resection (EOR) and OS with lung cancer BM after undergoing surgical resection; (2) evaluate the relationship among ALK and EGFR mutations with the rate of gross total resection (GTR), local control, and OS; and (3) discuss the targeted therapies administered for mutational lung cancer management and their potential role in local control and OS.

## 2. Materials and Methods

### 2.1. Study Design

This surgical series was a multi-site, retrospective study of all cerebral lung cancer patients undergoing resection at Emory University Healthcare hospital between January 2012 and May 2022, including two tertiary, academic referral hospitals, and two mid-sized, community hospitals. We collected data from the CNS Tumor Outcomes Registry at Emory (CTORE), a prospectively managed patient outcomes database for central nervous system (CNS) tumors treated at participating sites. Our study was approved by the institutional review board at Emory University and had obtained an informed consent waiver.

### 2.2. Patient Selection and Data Collection

Patients undergoing surgical resection for BM with a diagnosis of primary lung cancer within the study period were included. Eligible patients were identified from the CTORE database and data were collected from primary review of the electronic medical record, including clinical, pathology/genomic, and imaging data. These were reviewed for demographic data, Karnofsky Performance Scale (KPS), surgical procedure details and frequency including EOR, complications (i.e., motor weakness or speech trouble after surgery, wound infection, CSF leak, and/or seizures), local recurrence, pathology (i.e., primary tumor and mutational statuses), extent of systemic disease (i.e., disease in bone, adrenal glands, liver or other organs) and control of disease, prior therapy (i.e., chemotherapy, radiation, and/or immunotherapy), adjuvant/postoperative therapy, and discharge/readmission information. Imaging data were used for BM characterization, including location, number of metastatic lesions, and volume of dominant lesion. Data regarding preoperative as well as postoperative targeted therapy and radiation were also collected. The type of radiation administered was characterized as either standard-fractionated, WBRT, or SRS. Genomic data were obtained from pathology reports and Caris molecular profiling (Caris Life Sciences, Dallas, TX, USA). Patient driver mutational statuses were categorized as either EGFR-amplified, ALK-rearranged mutations present in the biopsied brain tissue, or neither.

### 2.3. Statistical Analysis

Statistical analysis was performed using SAS version 9.4 (SAS Institute, Cary, NC, USA). Descriptive statistics were performed for all demographic data. The primary end points of the study were OS and local recurrence free survival (LRFS). OS was defined as the time from the date of surgery to the date of death or the date of last follow-up. LRFS was defined as the time from the date of surgery to the date of local recurrence or the date of last follow-up. Univariate and multivariable analyses were performed by Cox proportional hazard models to assess factors associated with OS and LRFS. Multivariable models were selected using the backward variable selection approach with an alpha of removal of 0.2. To account for the small sample size and low event rates, Firth’s penalized likelihood bias-reduction approach was used. The Kaplan–Meier method was used to generate OS and LRFS curves and survival curves were compared between different groups using the log-rank test. Statistical significance was set at an alpha value of 0.05.

## 3. Results

### 3.1. Patient Population

During the study period between January 2012 and May 2022, 95 patients (Table 1) underwent surgical resection of cerebral lung cancer with accessible molecular testing of the biopsied brain tissue and were available to review in our electronic medical record. This patient cohort had a mean age of 63.2 ± 10.4 years with 54 (56.8%) female patients and 41 (43.2%) male patients. Median preoperative KPS for the patient cohort was 80 (Table 1). A total of 43 (45.3%) patients had local recurrence of the resection site post-surgery. A total of 7 (7.4%) patients had ALK-rearranged mutations and 19 (20.0%) patients had EGFR-amplified mutations. The mean number of metastatic brain lesions for patients was 2.64 ± 2.97 prior to surgery and the median number of metastatic lesions that developed post-surgery was 1 (0–3). The median time from lung cancer diagnosis to BM was 244 (12–932) days. A total of 72 (79.1%) patients had GTR of the BM during their first craniotomy. Six patients required reoperation for BM resection and, of those, 83.3% of patients had GTR. A total of 54 (58.7%) patients were readmitted after discharge with presentations that included hypotension, tachycardia, seizures, altered mental status, speech and motor impairments, and disease worsening (Table 2).

In our study, three (42.9%) patients with ALK mutations (Table 1) received targeted therapy in the form of TKIs and had a mean OS of 0.97 years. Of these patients, three (42.9%) had adjuvant targeted therapies in the form of Crizotinib (TKI), Alectinib (TKI), and Lorlatinib (TKI). Two (28.6%) ALK patients had targeted TKI therapy prior to surgical resection in the form of Crizotinib with a mean OS of 2.93 years prior to requiring surgical intervention.

Furthermore, eight (42.1%) patients with EGFR mutations (Table 1) received targeted therapy in the form of TKIs and mAbs and had a mean OS of 3.06 years after surgical resection of BMs. Of these patients, eight (88.9%) had adjuvant targeted therapies in the form of Atezolizumab (mAb), Osimertinib (TKI), Pembrolizumab (mAb), and Erlotinib (TKI). Six (31.6%) patients had targeted therapy prior to surgical resection in the form of Gefitinib (TKI), Erlotinib, and Osimertinib with a mean OS of 4.39 years prior to requiring surgical intervention.

In addition, 30 (31.6%) patients without records of ALK or EGFR mutations in their biopsied brain tissue received immunotherapy (Table 1) and had a mean OS of 2.50 years. Of these patients, 21 (70.0%) had adjuvant targeted therapies in the form of Atezolizumab, Ipilimumab (mAb), Pembrolizumab, Nivolumab (mAb), Ramucirumab (mAb), and Erlotinib. Ten (33.3%) patients had immunotherapy prior to surgical resection in the form of Durvalumab, Nivolumab, Atezolizumab, Pembrolizumab, Crizotinib, and Denosumab (mAb) with a mean OS of 1.57 years prior to requiring surgical intervention.

**Figure 1:**
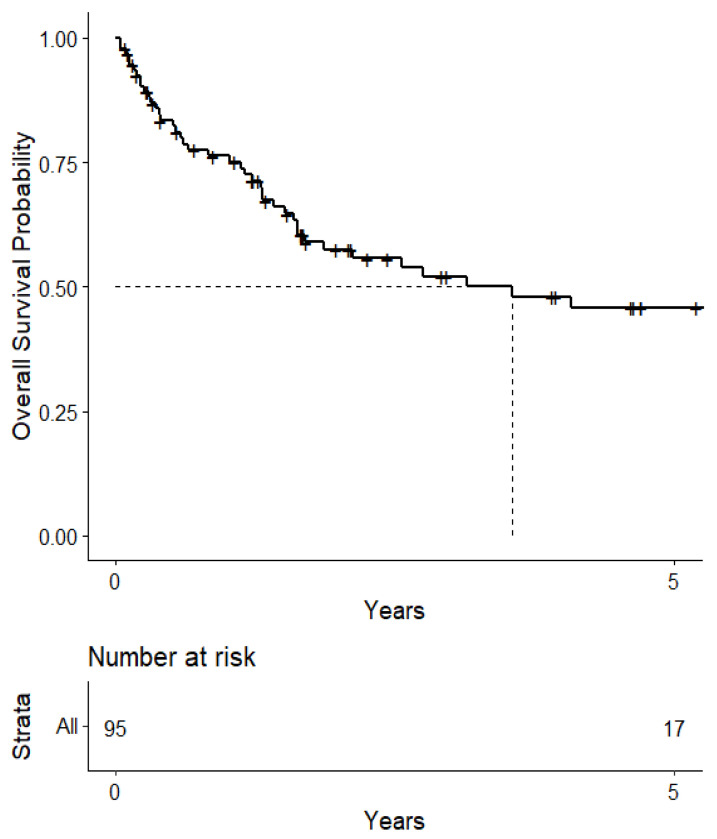
Kaplan–Meier curve of survival from 1st treatment administration.

**Figure 2:**
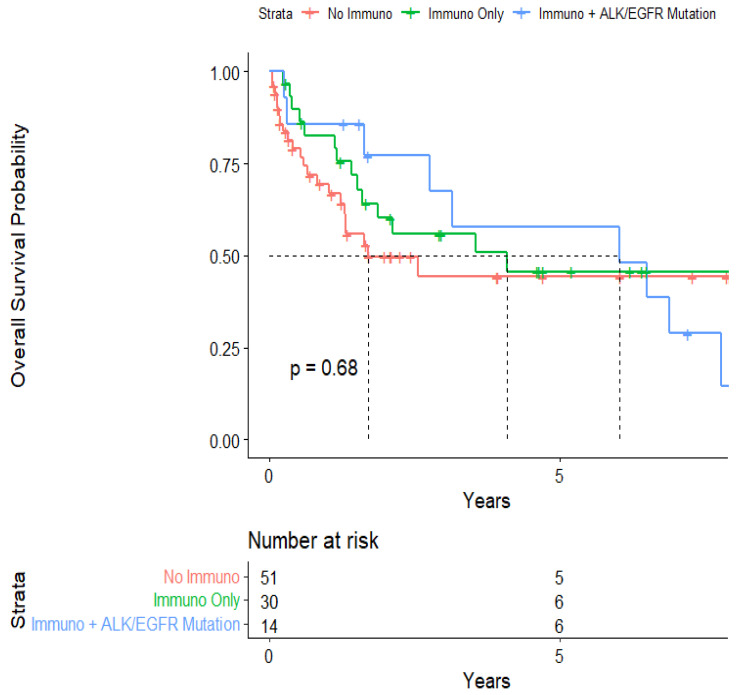
Kaplan–Meier curve of survival from 1st treatment administration stratified by immunotherapy and mutational status.

The median survival from the start of treatment administration was 3.55 (95% CI: 1.70-NA) years (Figure 1). When stratified, the median survival was 6.01 (95% CI: 2.75-NA) years for patients who had immunotherapy for their NSCLC and an ALK or EGFR mutation that required targeted therapy in the form of TKIs and mAbs. On the other hand, the median survival from the start of treatment administration was 4.08 (95% CI: 1.59-NA) years for patients who had immunotherapy for their NSCLC but not ALK or EGFR mutation-related targeted therapy, and 1.70 (95% CI: 1.30-NA) years for patients who did not have immunotherapy or ALK or EGFR mutation-related targeted therapy (Figure 2). These levels were not statistically significant from each other, however.

### 3.2. Univariate Association with Overall Survival and Local Recurrence-Free Survival

Univariate analysis of our surgical cohort found adjuvant radiation therapy (HR: 0.26; 95% CI: 0.12–0.52; *p*-value = <0.001), adjuvant chemotherapy (HR: 0.54; 95% CI: 0.30–0.98; *p*-value = 0.040), new brain metastases within a year of surgery (HR: 3.37; 95% CI: 1.66–6.85; *p*-value = <0.001), systemic progressive disease (HR: 3.82; 95% CI: 1.84–7.92; *p*-value = <0.001), and type of radiation therapy (SRS vs. standard fractionated) (HR: 0.44; 95% CI: 0.20–0.94; *p*-value = 0.030) as prognostic factors for overall survival (Table 3). ALK-rearranged (HR: 2.26; 95% CI: 0.87–5.84; *p*-value = 0.085) and EGFR-amplified (HR: 1.63; 95% CI: 0.76–3.49; *p*-value = 0.692) mutations were not significantly associated with overall survival. Furthermore, there was no statistical difference in OS between patients with GTR versus near-total resection.

In addition, univariate analysis of our surgical cohort found number of new brain metastases within a year of surgery (HR: 1.07; 95% CI: 1.01–1.14; *p*-value = 0.030) and volume of tumor resected in the first craniotomy (HR: 1.02; 95% CI: 1.00–1.03; *p*-value = 0.024) as prognostic factors for LRFS (Table 4). EGFR/ALK mutations and EOR were not significant prognostic factors for LRFS.

**Figure 3:**
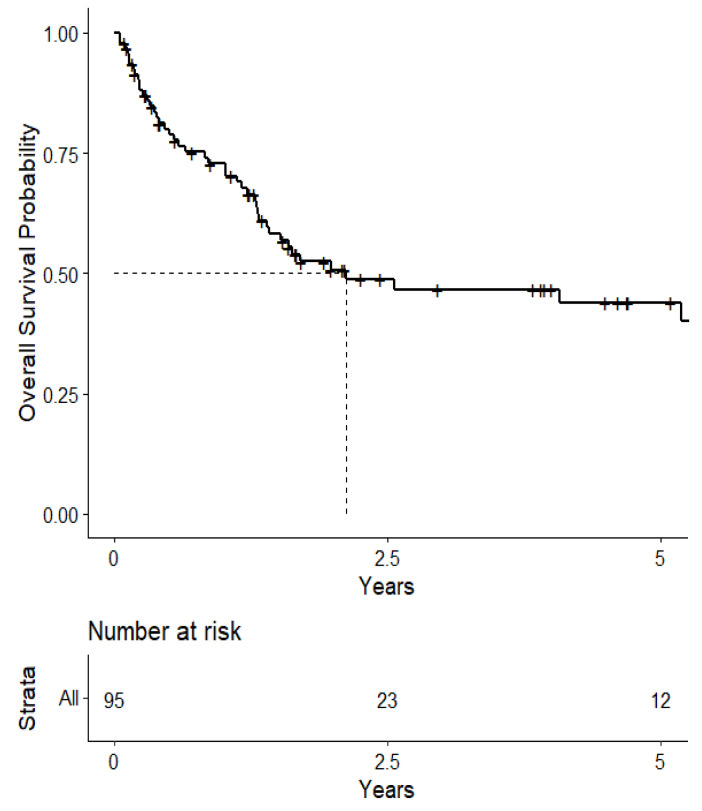
Kaplan–Meier curve of overall survival.

**Figure 4:**
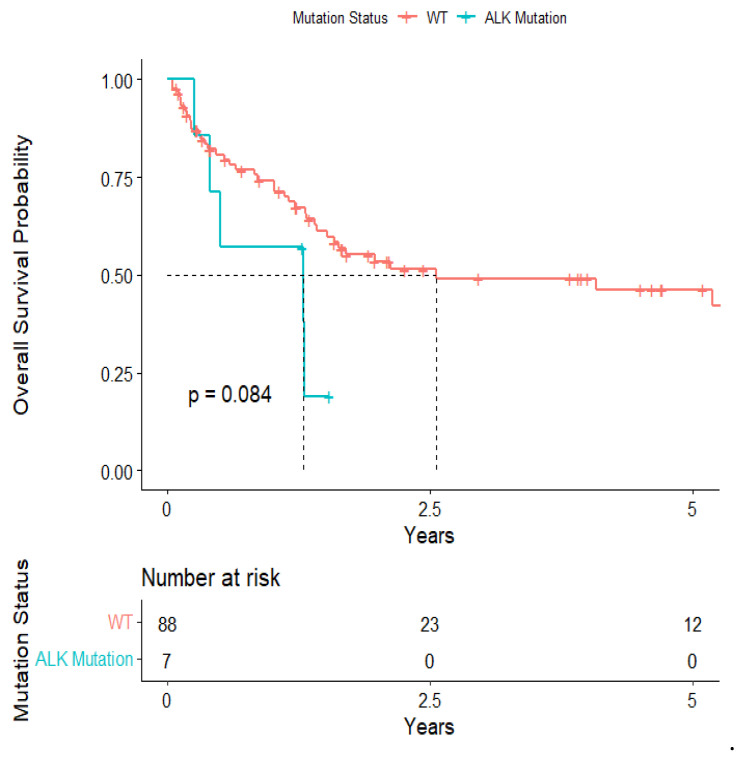
Kaplan–Meier survival curve of ALK-rearranged mutational status predicting overall survival.

**Figure 5:**
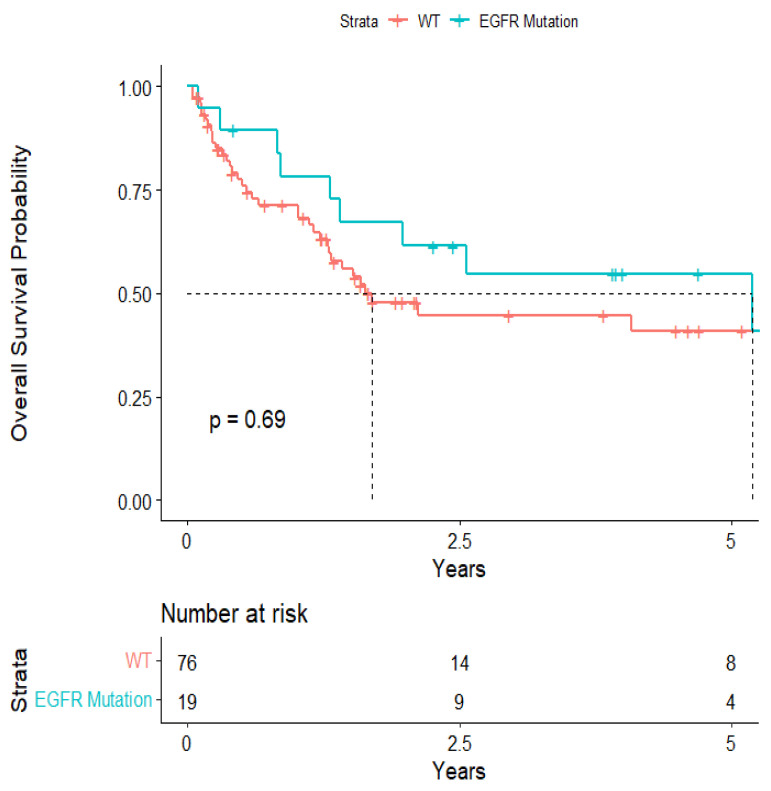
Kaplan–Meier survival curve of EGFR-amplified mutational status predicting overall survival.

**Figure 6:**
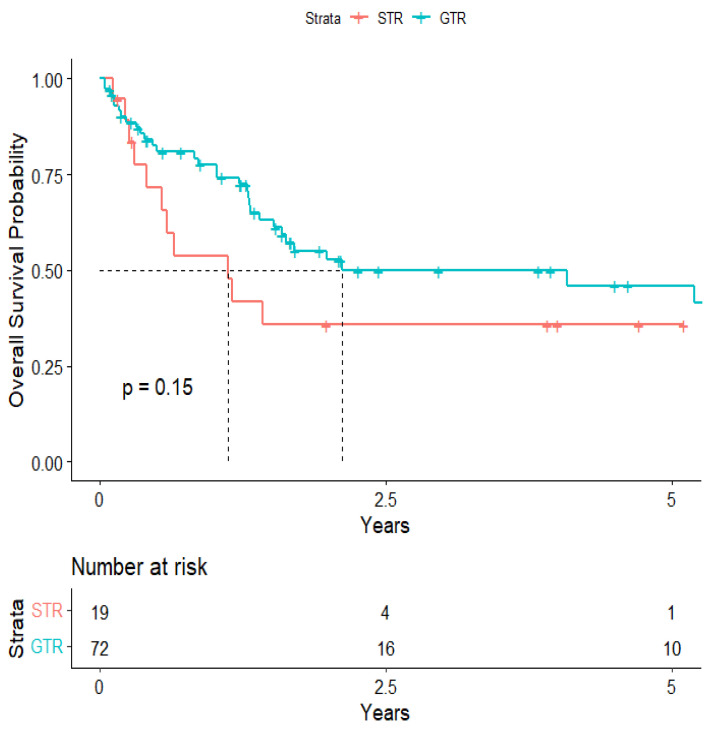
Kaplan–Meier curve of extent of resection from 1st craniotomy predicting overall survival.

**Figure 7:**
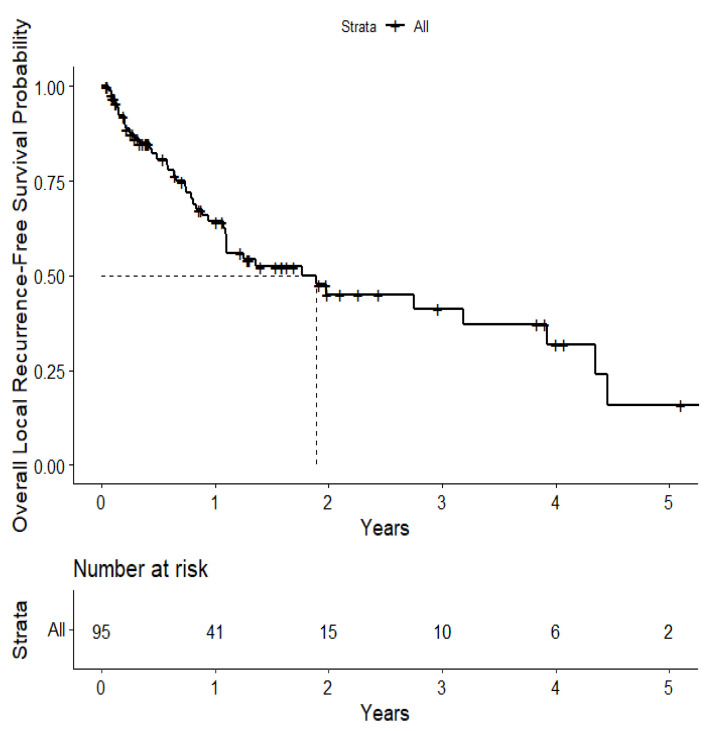
Kaplan–Meier curve of local recurrence-free survival.

**Figure 8:**
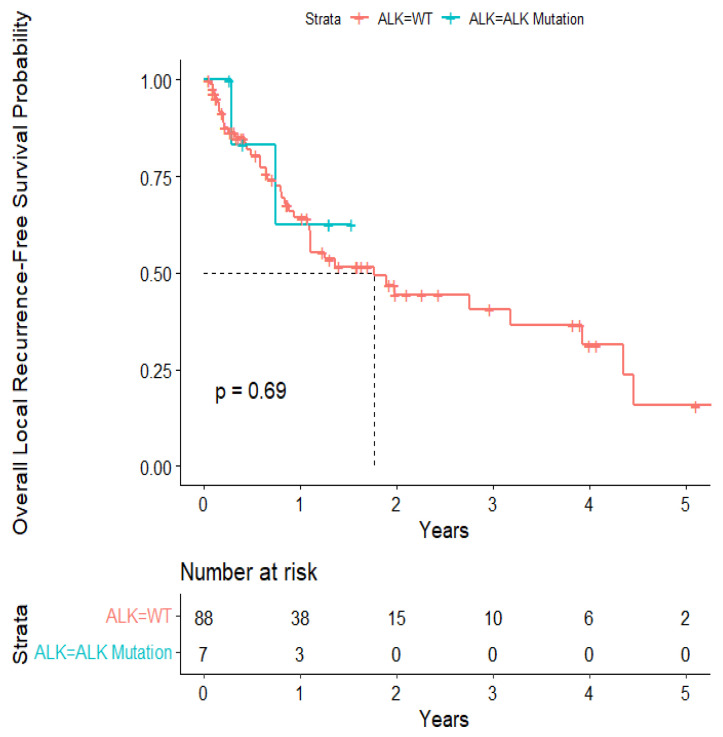
Kaplan–Meir survival curve of ALK-rearranged mutational status predicting local recurrence-free survival.

**Figure 9:**
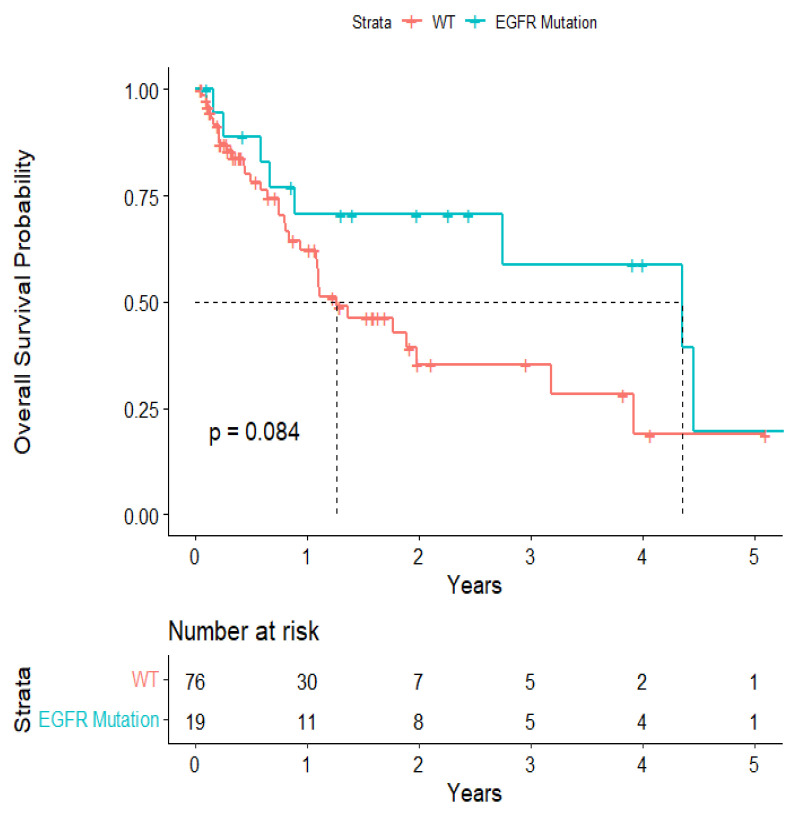
Kaplan–Meir survival curve of EGFR-rearranged mutational status predicting local recurrence-free survival.

**Figure 10:**
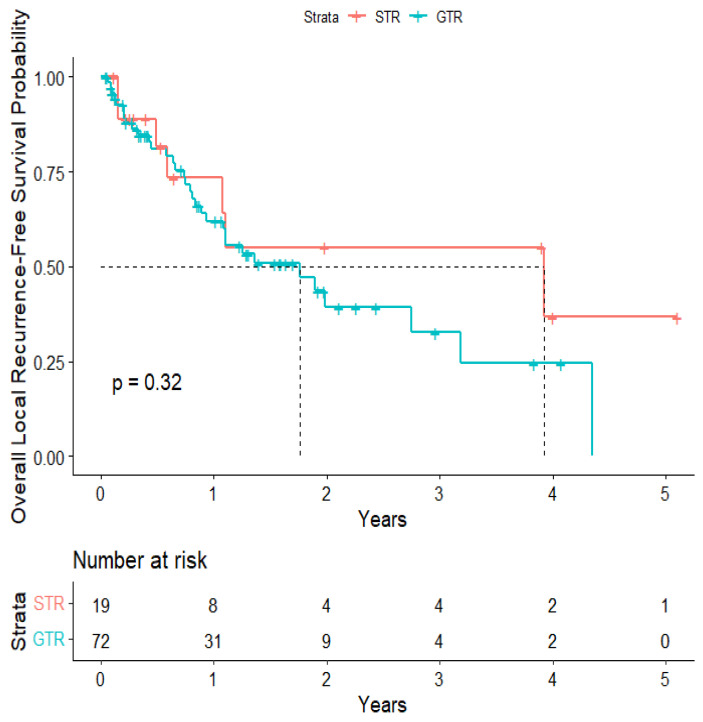
Kaplan–Meier curve of extent of resection from 1st craniotomy predicting local recurrence-free survival.

The overall median survival was 2.12 years (Figure 3). The median survival for patients with ALK-rearranged mutations was 1.29 years (Figure 4), and the median survival for patients with EGFR-amplified mutations was 5.19 years (Figure 5). Additionally, the median survival for patients with GTR was 2.12 years (Figure 6). Furthermore, the overall LRFS was 1.89 years (Figure 7). Median LRFS could not be calculated for ALK patients due to limited sample size (Figure 8). The median LRFS for EGFR patients was 4.35 years (Figure 9). The median LRFS for patients with GTR was 1.76 years (Figure 10).

### 3.3. Multivariate Associations

For OS, systemic progressive disease (HR: 4.74; 95% CI: 1.89–11.85; *p*-value ≤ 0.013), type of radiation therapy (SRS vs. standard fractionated) (HR: 0.30; 95% CI: 0.14–0.68; *p*-value = 0.008), new brain metastases within a year of surgery (HR: 1.12; 95% CI: 1.01–1.23; *p*-value = 0.010), and adjuvant chemotherapy (HR: 0.46; 95% CI: 0.21–1.02; *p*-value = 0.024) were prognostic factors for survival (Table 5).

## 4. Discussion

With the growing importance of mutational marker analysis to target therapy, the multimodal management of BMs is poised for significant change. Consequently, multidisciplinary teams of neurosurgical, neuro-oncological, and radiation practitioners must be aware of the effects that mutations play on OS, systemic treatment response, and, potentially, regarding intracranial disease control as well. Our retrospective study evaluates the impact of ALK and EGFR mutational markers in the management, local control, and EOR of BMs as well as by discussing the potential role that newer second-line targeted therapies played for BM management.

Additionally, 30 patients without ALK or EGFR mutations in the biopsied brain tissue received targeted therapies, but they did not have ALK or EGFR mutations in the biopsied brain tissue. This may be attributed to ALK and EGFR mutations in the primary lung tumor sites that were not present in BM pathology. Such an incoherence between primary tumor and metastatic lesion mutational status is known as discordance. Discordance is common among metastatic lung cancer patients, with 30–50% of cases reporting this phenomenon [42,43]. As a result, outcomes of targeted therapy are further complicated, as discordant tumors could act as confounding factors on survival and the overall efficacy of treatments [44,45].

Our multivariate analysis showed that systemic progressive disease, type of radiation therapy, new BMs within a year of surgery, and adjuvant chemotherapy were prognostic factors for survival. These findings are externally validated by previous surgical series [46,47,48,49]. New BMs within a year of surgery is an especially interesting finding since current scientific literature does not provide a definitive time frame in which new BMs could yield a hazard to patient survival. Our analysis also showed that any new BMs within a year of resection can significantly reduce patient survival. Additionally, we also found the number of new BMs within a year of surgery and volume of tumor to be significant prognostic factors for LRFS. Tumor size has been a known predictor for local recurrence of BM and our results are externally validated by the literature [50,51]. The number of new BMs that occur within a year was another interesting prognostic factor. More new BMs meant a greater hazard for local recurrence of the disease, a finding that, although consistent with previous literature, is new and more specific [52,53].

Some of our results, however, had differing findings from sources in the external literature that studied the efficacy of BM mutational status and applied targeted therapy. Most notably, unlike the prior work by Colditz et al. detailing a relationship between BRAF mutational status predicting favorable outcomes in melanoma patients [41], we were not able to find significant associations between mutational status and EOR as well as OS in our lung cancer cohort. Their study cited heterogeneous treatment protocols, where first-line (single-agent immune checkpoint therapy) and second-line (anti-CTLA-4 and anti-PD-1) therapies were used at various time periods. Furthermore, their institution also had limitations on which patients could access BRAF-targeted therapy, requiring treatment with immune-checkpoint therapy first. Thus, varying administration protocols create an unstandardized basis for comparing our results to those of Colditz et al. Furthermore, the differences in our findings can also be attributed to potential deviations in the mechanisms of the pathophysiological disease progression to BM between metastatic lung cancer and melanoma.

Furthermore, our ALK and EGFR mutational patient cohort that received targeted therapy had a larger median OS and LRFS (6.01 and 5.75 years, respectively) than the group that did not have any mutations in biopsied brain tissue but did receive immunotherapy (4.08 and 1.89 years, respectively). These survival times, however, were greater than the median survival time for patients who did not receive any immunotherapy (1.70 and 1.76 years, respectively). The cost versus the benefit of improving OS by this much should be assessed in future studies. All three of our patient cohort groups had longer survival times than those published in the literature, especially for the groups that received targeted therapies, which tended to be 8–26 months OS [54,55,56,57,58]. In addition, literature findings showed 18–24 months to be the range for local recurrence free survival, externally validating our findings [59,60,61,62]. Furthermore, in our cohort, EGFR mutational status trended towards being significant in prolonging LRFS, which is in line with findings in the literature as well [63]. Thus, the combinations of targeted therapies in this series and their respective chronology of use seem to confer survival and local control benefits and will be studied further through future work from our group to create and evaluate formally assessable results.

### Limitations

Our study was limited by its retrospective nature. Although targeted therapies for ALK-rearranged and EGFR-amplified mutations show promise in combination with surgical resection of cerebral lung cancer, additional analysis and further studies must elucidate these relationships to optimize their inclusion in treatment protocol. Larger sample sizes and randomized studies could yield better associations between surgical resection, mutation status, targeted therapy, and postoperative outcomes. Furthermore, our study spans 10 years; during this time, there has been an evolution of targeted therapies used for ALK-rearranged and EGFR-amplified mutations. Future work will focus on improved follow-up and increased numbers of patients via multi-institutional collaboration with complete genomic sequencing as well as classifying the patient mutational status and their respective therpies at a more granular level of detail. Given the time period of our studies, we also expect increasing utilization of cerebral-penetrant systemic therapies in future patients that will allow us to better evaluate the role of these therapies in surgical BM patients.

## 5. Conclusions

With our retrospective review of 95 patients undergoing surgical resection of cerebral lung cancer from January 2012 to May 2022, we identified key prognostic factors for OS and LRFS. Additionally, we assessed the role of ALK and EGFR mutations in local control of disease and OS. Patients with either ALK or EGFR mutation-related targeted therapies combined with immunotherapy showed the highest survival, though differences between groups were not statistically significant. Prognostic factors for OS included systemic progressive disease, type of radiation therapy used, new BMs within a year of surgery, and adjuvant chemotherapy.

In the evolving landscape of genomic medicine, our findings underscore the significance of directed-oncological therapies. We hope to continue building upon our work as we gather more patients who have received second line CNS-penetrant therapies better able to cross the blood–brain barrier and conduct follow-up studies comparing subsequent therapy cohorts to traditional systemic therapy. Furthermore, we hope to raise awareness for the administration of combination targeted therapies as well as their timing to inspire future scientific investigations for these questions. Gaining a better understanding of these second-line therapies will enable individualized therapies to potentially increase survival times and local control of BM.

## Figures and Tables

**Table 1:** Clinical characteristics of cerebral lung cancer patients with pertinent demographic and surgical variables.

Variable	Level	*N* (%) = 95
Age	Mean (SD)	63.2 (10.4)
Gender	Male	41 (43.2)
KPS	Median (IQR)	80 (70–90)
ALK Mutation		7 (7.4)
	Received adjuvant targeted therapy	3 (42.9)
EGFR Mutation		19 (20.0)
	Received adjuvant targeted therapy	8 (42.1)
Patients without ALK/EGFR mutations receiving immunotherapy		30 (31.6)
Was brain mets 1st presentation		66 (69.5)
Disease in bone		28 (29.5)
Disease in adrenal gland		13 (13.7)
Disease in liver		12 (12.6)
Prior chemotherapy		41 (43.2)
Prior immunotherapy		10 (10.5)
Prior radiation		36 (37.9)
Additional radiation given		34 (42.5)
Lobe of brain	Frontal	35 (36.8)
	Temporal	6 (6.3)
	Parietal	22 (23.2)
	Occipital	6 (6.3)
	Cerebellar	26 (27.4)
Number of metastatic brain lesions	Mean (SD)	2.64 (2.97)
Number of new brain metastases post-surgery	Median (IQR)	1 (0–3)
Volume of dominant brain mets, cm^3^	Mean (SD)	17.28 (19.37)
Time to brain mets from lung cancer diagnosis (days)	Median (IQR)	244 (12–932)
Extent of resection—1st craniotomy	Gross total resection	72 (79.1)
Extent of resection—2nd craniotomy	Gross total resection	5 (83.3)

**Table 2:** Complications of cerebral lung cancer patients with pertinent outcome variables.

Variable	*N* (%) = 95
Wound infection after surgery	3 (3.2)
CSF leak after surgery	1 (1.1)
Intracerebral hemorrhage	2 (2.1)
Seizures after surgery	4 (4.2)
Local recurrence	43 (45.3)
Readmission	54 (56.8)
Readmission within 30 days	30 (31.6)
Reoperation required	6 (6.4)

**Table 3:** Univariate association with predictors of overall survival.

Covariate	Level	*N*	OS (yrs)	
			Hazard Ratio (95% CI)	*p*-Value
Adjuvant radiation therapy		81	0.26 (0.12–0.52)	**<0.001**
ALK mutation		7	2.26 (0.87–5.84)	0.085
EGFR mutation		19	1.63 (0.76–3.49)	0.692
Adjuvant chemotherapy		54	0.54 (0.30–0.98)	**0.040**
Previous radiation		36	1.04 (0.58–1.89)	0.889
Motor weakness or speech trouble after surgery		31	1.68 (0.93–3.02)	0.081
Number of new brain metastases within a year of Ssurgery			1.03 (0.98–1.08)	0.281
New brain metastases within a year of surgery			3.37 (1.66–6.85)	**<0.001**
Systemic progressive disease		54	3.82 (1.84–7.92)	**<0.001**
Type of radiation therapy	SRS (1, 3, or 5 fractions)	66	0.44 (0.20–0.94)	**0.030**
	Standard fractionated WBrT (10 or 15 fractions)	15	-	-
Volume of tumor—1st carniotomy			1.01 (0.99–1.02)	0.304
Extent of resection—1st surgery	Gross-total resection	72	0.60 (0.30–1.21)	0.154
	Subtotal resection	19	-	-

**Table 4:** Univariate association with predictors of local recurrence-free survival.

Covariate	Level	*N*	LRFS (yrs)	
			Hazard Ratio (95% CI)	*p*-Value
Adjuvant radiation therapy		81	4.86 (0.66–35.76)	0.087
ALK mutation		7	0.75 (0.18–3.13)	0.691
EGFR mutation		19	0.50 (0.23–1.11)	0.084
Adjuvant chemotherapy		54	0.74 (0.38–1.44)	0.372
Previous radiation		36	0.57 (0.29–1.11)	0.093
Motor weakness or speech trouble after surgery		31	1.34 (0.69–2.62)	0.386
Number of new brain metastases within a year of surgery			1.07 (1.01–1.14)	**0.030**
New brain metastases within a year of surgery		45	1.06 (0.96–1.19)	0.253
Progressive disease		54	1.37 (0.74–2.54)	0.311
Type of radiation therapy	SRS (1, 3, or 5 fractions)	66	1.30 (0.54–3.14)	0.558
	Standard fractionated WBrT (10 or 15 fractions)	15	-	-
Volume of tumor—1st craniotomy			1.02 (1.00–1.03)	**0.016**
Extent of resection—1st surgery	Gross-total resection		1.53 (0.66–3.53)	0.316
	Subtotal resection		-	-

**Table 5:** Multivariate association with predictors of overall survival.

Covariate	Level	OS (yrs)		
		Hazard Ratio (95% CI)	Adjusted *p*-Value	Model *p*-Value
				**<0.001**
Systemic progressive disease		4.74 (1.89–11.85)	**0.013**	-
Type of radiation therapy	SRS (1, 3, or 5 fractions)	0.30 (0.14–0.68)	**0.008**	-
	Standard fractionated WBrT (10 or 15 fractions)	-	-	-
New brain metastases within a year of surgery		1.12 (1.01–1.23)	**0.010**	-
Adjuvant chemotherapy		0.46 (0.21–1.02)	**0.024**	-

## Data Availability

Due to privacy and ethical restrictions, the patient data supporting the reported results in this study cannot be shared publicly. Access to the anonymized data may be granted upon reasonable request and subject to appropriate ethical approval.
